# Non-Cellulosic Polysaccharides from Cotton Fibre Are Differently Impacted by Textile Processing

**DOI:** 10.1371/journal.pone.0115150

**Published:** 2014-12-17

**Authors:** Jean-Luc Runavot, Xiaoyuan Guo, William G. T. Willats, J. Paul Knox, Florence Goubet, Frank Meulewaeter

**Affiliations:** 1 Bayer CropScience N.V., Innovation Center, Technologiepark 38, Gent, Belgium; 2 Department of Plant and Environmental Sciences, University of Copenhagen, 1871, Frederiksberg, Denmark; 3 Centre for Plant Sciences, Faculty of Biological Sciences, University of Leeds, Leeds, LS2 9JT, United Kingdom; INRA, France

## Abstract

Cotton fibre is mainly composed of cellulose, although non-cellulosic polysaccharides play key roles during fibre development and are still present in the harvested fibre. This study aimed at determining the fate of non-cellulosic polysaccharides during cotton textile processing. We analyzed non-cellulosic cotton fibre polysaccharides during different steps of cotton textile processing using GC-MS, HPLC and comprehensive microarray polymer profiling to obtain monosaccharide and polysaccharide amounts and linkage compositions. Additionally, *in situ* detection was used to obtain information on polysaccharide localization and accessibility. We show that pectic and hemicellulosic polysaccharide levels decrease during cotton textile processing and that some processing steps have more impact than others. Pectins and arabinose-containing polysaccharides are strongly impacted by the chemical treatments, with most being removed during bleaching and scouring. However, some forms of pectin are more resistant than others. Xylan and xyloglucan are affected in later processing steps and to a lesser extent, whereas callose showed a strong resistance to the chemical processing steps. This study shows that non-cellulosic polysaccharides are differently impacted by the treatments used in cotton textile processing with some hemicelluloses and callose being resistant to these harsh treatments.

## Introduction

Cotton (*Gossypium sp*) fibre has been widely studied as the major natural fibre used in the textile industry and as an excellent model for fibre development [1], [2]. Cotton fibre development goes through four, partially overlapping, phases: fibre initiation, elongation, secondary thickening, and maturation [1], [3]. During these different stages, the various polysaccharides that will constitute the mature fibre are produced. The fibre primary cell wall is formed during the fibre initiation and elongation stages and is mainly composed of pectins, cellulose, and hemicelluloses [1], [4]–[6]. The non-cellulosic polysaccharides have been widely studied during fibre initiation [7]– or elongation [4], [5], [10], [11] to understand their roles in the fibre construction and development. When cotton fibre is at 15 to 19 dpa, secondary cell wall synthesis starts resulting in the deposition of large amounts of cellulose to finally reach around 95% of total mass in the mature cotton fibre [12].

As cellulose is the major component of the cotton fibre, the fate of the other polysaccharides during textile processing has not been studied so far. Due to the harsh treatments that occur during textile processing it is widely assumed in the literature that the processed cotton primarily consists of cellulose and that the non-cellulosic polysaccharides are removed [13]. Textile processing is basically composed of three mechanical steps and several chemical treatments depending on the desired finishing [14], [15]. The fibres are first organized into slivers, these slivers undergo spinning to produce yarn that will finally be knitted or woven into fabric. The cotton then undergoes many different chemical treatments such as scouring (to remove impurities like seed fragments, pectins and natural wax), bleaching (to improve fibre whiteness), mercerizing (to improve lustre, strength and dye affinity), dyeing, or finishing treatments. The finishing treatments are meant to produce textiles with various added values (softness, water repellency, flame retardancy, …). From these treatments, scouring has been shown to remove pectins (and waxes) from cotton [16]. Many studies have been performed to improve the efficiency [17], [18], to shorten the time [16], [19] or to reduce the ecological impact [13] of the different chemical treatments. Recent literature has focused on the addition of other compounds to the fabrics [20] or on improving cellulose functionalization [21]–[23] to improve dyeability or other desired textile characteristics. In this study, we followed the non-cellulosic polysaccharides during the steps of textile processing to determine whether they are all removed as it is currently assumed or if some of them are more resistant to the different mechanical/chemical treatments. We applied different biochemical techniques on industrially produced cotton samples to determine the impact of the different textile processing steps on polysaccharide composition. Several of the non-cellulosic polysaccharides, such as xylan, xyloglucan and callose, appeared to be (partly) retained during textile processing.

## Materials and Methods

### Material

Cotton fibre, yarn, raw and treated fabrics samples from the same industrial processing chain were kindly provided by Utexbel NV (Belgium). Prior to analysis, cotton fabrics were first deknitted into yarn and yarn was frayed to obtain unraveled fibres. Before the biochemical assays, the raw fibres and the unraveled fibres were subjected to a fine grinding with a liquid nitrogen-cooled crusher (SPEX Sample Prep Freezer/mill 6870, United Kingdom) to increase homogeneity and to maximize the extraction of polysaccharides. Powder was stored at −20°C to be used for all the experiments. All chemicals used for the analyses were purchased from Sigma-Aldrich.

### Textile processing conditions

Cotton fibres (Fib) were first organized into slivers, spun to produce yarn (Y), and knitted to obtain the raw fabric (RF). This raw fabric was washed for 20 min at 60°C with 2 g.L^−1^ of non-ionogenic washing product (Felosan JET), followed by an alkaline scouring and bleaching during 15 min at 100°C using 3 g.L^−1^ of 33% NaOH, 4% of 35% H_2_O_2_, 2 g.L^−1^ of non-ionogenic washing product (Felosan JET), and 2 g.L^−1^ of anion-active bleaching stabilizer (Contavan TIG50) (referred to as bleached/scoured fabric, B). Mercerization was achieved by applying 15% NaOH for a few minutes (mercerized fabric, M). The samples were then rinsed with water and the pH neutralised with acetic acid to obtain the ready-to-dye fabric (R). This fabric was then subjected to dyeing with 22.5 g.L^−1^ of Red Remazol RB 133, 0.38 g.L^−1^ of Brilliant Blue Remazol BB 133 and 6.7 g.L^−1^ of Yellow Remazol R in the presence of 30 g.L^−1^ of Na_2_CO_3_ and 25 g.L^−1^ of NaOH 18%. The dyed fabric received a finishing treatment with 15 g.L^−1^ of polyvinylacetate and 5 g.L^−1^ of polyethylene softener (POLYAVIN PEN), followed by drying at 160°C (finished fabric, F).

### Preparation of alditol acetates

Monosaccharide derivatization was carried out based on several alditol acetate protocols [24]–[26]. Briefly, 30 mg of powdered samples, to which 250 µg of internal standard (myo-inositol) had been added, were hydrolyzed in 1 mL of 2 M TFA for 2 h at 120°C. Hydrolyzed samples were dried and reduced in 100 µL of 50 mg.mL^−1^ sodium borohydride in 3 M ammonia for 1 h at 40°C. Excess borohydride was destroyed with 2 times 50 µL acetic acid and acetates were produced by adding 2 mL of acetic anhydride and 200 µL of 1-methylimidazole at room temperature for 20 min. The reaction was stopped by addition of 5 mL of water and partitioning by the addition of 2 mL of chloroform. After washing of the chloroform phase with 5 mL of water the chloroform was transferred to a vial for injection on a gas chromatograph (GC). Cellulose, being a very resistant polysaccharide, is only partly analysed by the methods used in this study. The results always refer to the extractible/accessible/hydrolysable polysaccharides that we considered as representative of the cotton fibres. This analysis detected neutral sugars only.

### Partial methylation for linkage analysis

Partial methylation was based on Ciucanu and Kerek [27] and Pettolino *et al.* [26] protocols with adaptations due to the high amount of cellulose in the samples: prior to the DMSO solubilisation, 30 mg of sample was boiled in water and precipitated in 70% ethanol and then dried. Finely powdered NaOH (40–50 mg) was added to the sample and stirred for 1 h. Iodomethane was added in 3 steps (150 µL, 150 µL and 200 µL respectively) and excess iodine was removed by the addition of 1 mL of 0.1 M sodium thiosulfate [28]. Partially methylated carbohydrates were recovered in 1 mL of chloroform and washed with 1 mL of water before being dried. Samples were further analyzed with the alditol acetate protocol starting from the hydrolysis step. This analysis detected neutral sugars only.

### GC acquisition parameters

Sugars were injected using the on-column mode of injection on a Trace GC ultra with an ISQ single quadrupole GC-MS (Thermo Scientific, Belgium) and a flame ionization detector (FID) associated with a high polarity, bonded phase SP-2380 capillary column 30 m×0.25 mm×0.2 µm (Supelco, USA) using hydrogen as the carrier gas with a flow rate of 4 ml.min^−1^. The MS transfer line was set at 275°C with 70eV electron impact ionization mode and the data from 41 to 450 m/z was acquired. The alditol acetates were separated by setting the oven to an initial temperature of 100°C, held for 0.5 min, and then ramped at 120°C.min^−1^ to 207°C, then ramped at 5°C.min^−1^ to 220°C, then ramped at 3°C.min^−1^ to 234°C, then ramped at 120°C.min^−1^ to 275°C and held for 4 min. The partially methylated alditol acetates were separated by setting the oven to an initial temperature of 110°C, held for 2 min, and then ramped at 120°C.min^−1^ to 180°C, then ramped at 5°C.min^−1^ to 220°C, then ramped at 120°C.min^−1^ to 250°C and held for 3 min, then ramped at 120°C.min^−1^ to 275°C and held for 4 min.

### Uronic acid assay

Samples (60 mg) were first subjected to hydrolysis in 700 µL of 2 M TFA for 2 h at 120°C. The hydrolysed samples were dried and washed 2 times with 500 µL of methanol before resuspension in 500 µL of water and filtration on 0.2 µm filters. Separation was performed by a Dionex HPLC (Thermo Scientific, Belgium) on a Carbopac PA200 column with an ECD detector. Buffer A was 100 mM NaOH and buffer B was 100 mM NaOH containing 1 M Na-acetate. Elution gradient was as follow: start at 8% B and hold for 2 min, then ramp to 30% in 15 min, then ramp to 60% in 2 min and hold for 5 min before going back to 8% in 2 min and hold for 10 min. Both galacturonic acid and glucuronic acid were quantified using standard curves.

### Monosaccharide and linkage analyses

Quantitative analysis of alditol acetates and uronic acids were performed referring to standard curves and the internal standard. Semi-quantitative data were obtained for linkage analysis using the FID signal associated to the MS signal from partially methylated alditol acetates and normalizing results with the terminal glucose signal (fixed at a value of 100) to take into account the losses during extraction and methylation.

### Antibodies

Several monoclonal antibodies were used during the microarray and microscopy experiments: LM6 [29], recognizing (1→5)-α-arabinan; JIM13 [30] and JIM20 [31], specific to arabinogalactan proteins (AGP) and extensin, respectively; LM15 [32], LM24 [33] and LM25 [33] recognizing different xyloglucan (XG) epitopes; AX1 [34], specific to arabinoxylan; LM5 [35] to galactan; BS-400-2 [36], to (1→3)-β-D-glucan; JIM5 [35] and LM19 [37] recognizing homogalacturonan (HG) with different methylesterification levels. Callose antibody BS-400-2 was purchased from Biosupplies (Australia). Anti-rat-FITC (F1763) and anti-mouse-FITC (F6257) antibodies were purchased from Sigma-Aldrich.

### Comprehensive Microarray Polymer Profiling (CoMPP)

CoMPP analysis was performed as previously described by Singh *et al.* [5] with minor modifications. Cadoxen extraction was omitted because it is mainly used to extract cellulose which we did not aim to analyse. For each processing stage, three replicates of 10 mg of ground fibre were extracted with 300 µL of solvents and the supernatants from the three sample replicates were printed in four technical replicates and four dilutions (1∶2, 1∶6, 1∶18 and 1∶54 [v/v] dilutions), giving a total of 48 spots representing each processing stage for each extraction. A heat map was generated to display the relative intensity of each signal to the maximum signal observed within the data for each antibody as shown in the raw data (S1 Table). For the calculations, values were taken from the sodium hydroxide extraction except for pectins where CDTA (1,2-diaminocyclohexane-N,N,N,N-tetraacetic acid) extraction values were used.

### Preparation for microscopy and fluorescence imaging

Before use, the wax of the cotton fibres was removed by incubating fibres at room temperature with excess ethanol for 90 min and again for 90 min in refreshed ethanol. The fibres were then incubated with acetone for 60 min, followed by incubation in ether for 60 min. The fibres were then left to air-dry. Resin embedding and immunolabeling was done as described by Marcus *et al.* [38] and by Kljun *et al.* [39]. Immunolabeling could not be performed on dyed/finished fabric as the color interfered with fluorescence detection. Fluorescence imaging of fibres was performed with an Olympus BX61 microscope equipped with epifluorescence irradiation. Images were acquired with a Hamamatsu ORCA285 camera and managed with Improvision Volocity software.

### Statistical analysis

All the results presented were tested for overall significant difference using the non-parametric Kruskall and Wallis test and they were all significant at 5%. The statistical significance of the impact of individual treatments was determined by analyzing the differences between consecutive samples with both a parametric (Student) and a non-parametric (Mann-Whitney) test. Only the results that showed a significant difference in both tests were considered significantly different and displayed in the figures.

## Results

### Mechanical processing steps do not impact on polysaccharide content

**Figure 1:**
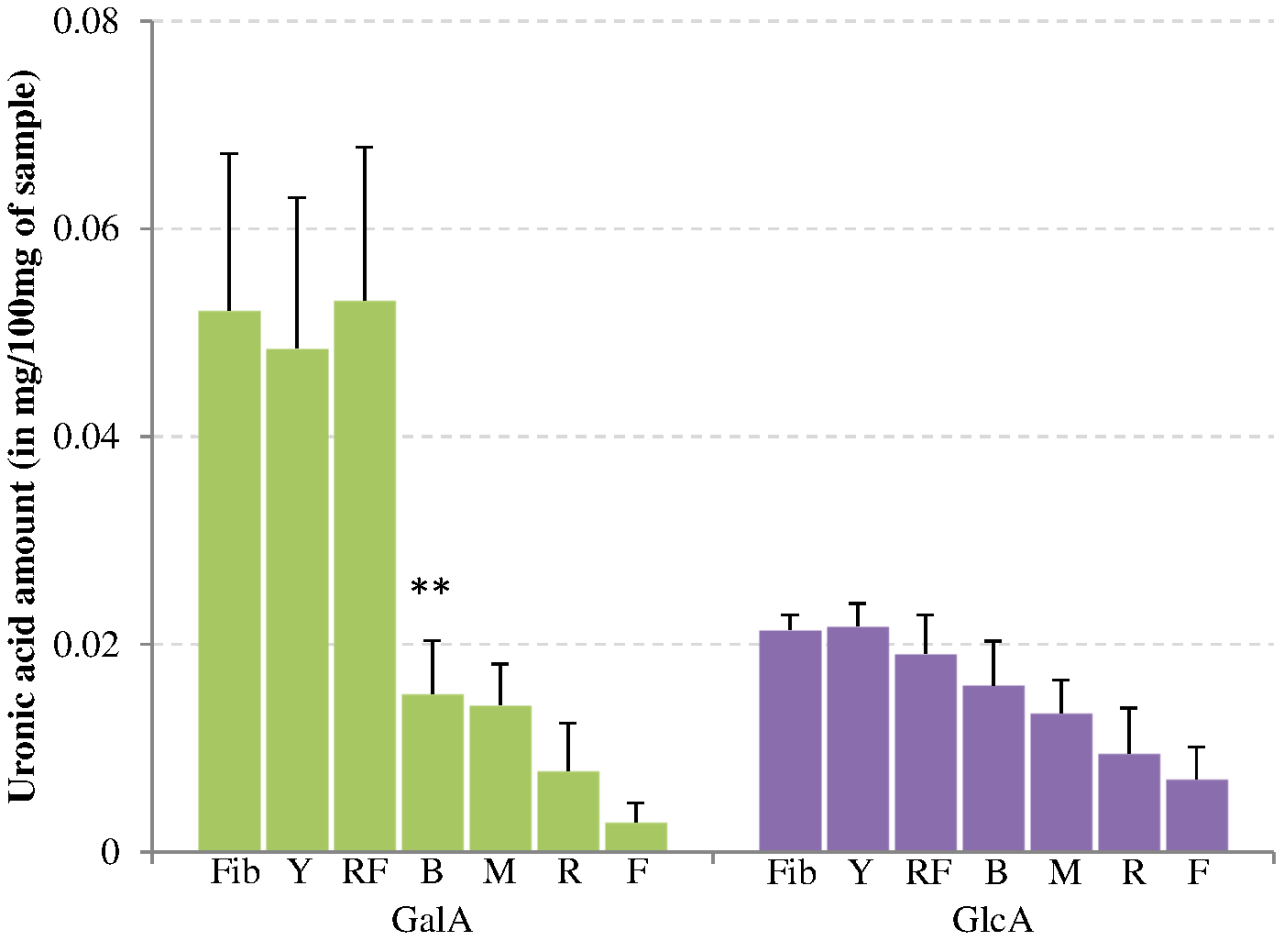
Uronic acid content of processed cotton samples. Extracted galacturonic acid (GalA) and glucuronic acid (GlcA) from powdered textile processing samples; Fib  =  fibre, Y =  yarn, RF =  raw fabric, B =  bleached/scoured fabric, M =  mercerized fabric, R =  ready-to-dye fabric, and F =  finished fabric. Error bars represent standard deviation (n = 3). ** indicates significant difference between bleached and raw fabric samples at p<0.01.

**Figure 2:**
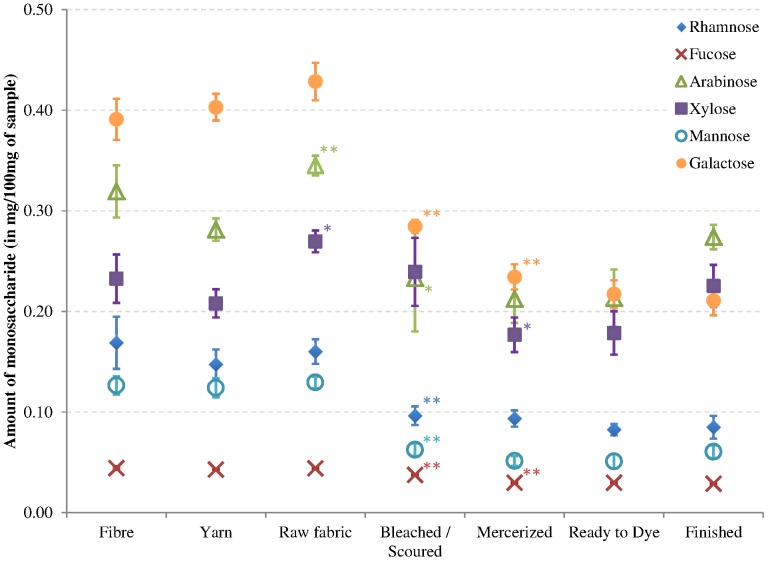
Amounts of individual neutral monosaccharides in cotton fibre and processed samples. Rhamnose (full diamond), fucose (cross), arabinose (full triangle), xylose (full square), mannose (empty circle) and galactose (full circle) were obtained after hydrolysis of powdered textile processing samples. Error bars represent standard deviation (n = 3). * and ** indicate significant difference between a sample and the preceding one at p<0.05 and p<0.01, respectively.

The first steps of textile processing consist of mechanical steps that convert the fibre to yarn and then to raw fabric. The data from Fig. 1 show that these steps do not lead to significant differences in the levels of acidic sugars. However, the levels of glucose and other neutral sugar levels do increase during these processing steps, with a significant increase being observed between the yarn and raw fabric (table 1). Assessment of individual neutral sugars (Fig. 2) indicated that there was a significant increase in arabinose and xylose in raw fabric compared to yarn but all of them have similar levels in the fabric compared to the fibre sample. These mechanical steps are thus not destroying or removing any polysaccharide and do not substantially change the chemical composition of the cotton. Therefore, for the other chemical analyses we chose to present the average values from these 3 stages as “untreated” samples that did not go through chemical treatments.

**Table 1:** Amounts of glucose and total neutral monosaccharides (except glucose) obtained after hydrolysis of powdered textile processing samples.

	Amount (mg per 100 mg of fibre)						
	Fibre	Yarn	Raw fabric	Bleached/Scoured	Mercerized	Ready to Dye	Finished
Glucose	**8.90**±1.74	**8.95**±1.42	**12.87** *±1.12	**11.50**±2.14	**11.20**±1.52	**11.78**±2.60	**13.55**±1.92
Neutral monosaccharides except glucose	**1.33**±0.09	**1.25**±0.06	**1.42** *±0.05	0.97**±0.13	**0.80**±0.06	**0.77**±0.08	**0.88**±0.07

Standard deviation are indicated (n = 3).

* and ** indicate significant difference between a sample and the preceding one at p<0.05 and p<0.01, respectively.

### Bleaching/scouring removes some polysaccharides

**Figure 3:**
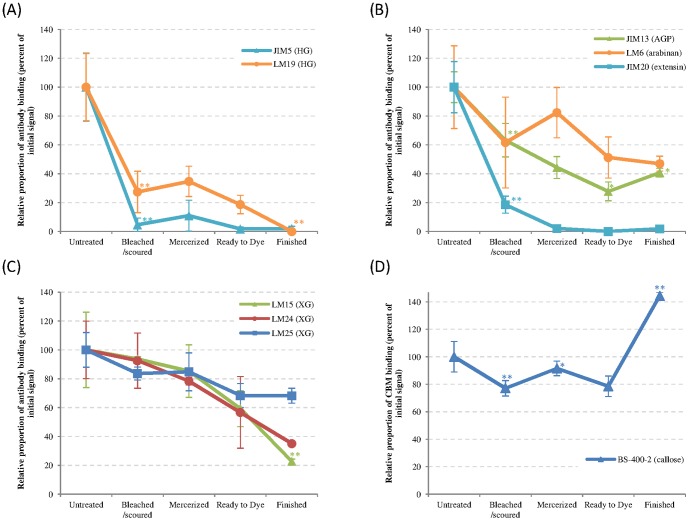
Comprehensive microarray polymer profiling of powdered textile processing samples. Detection of epitopes using the following probes: (a) Anti-homogalacturonan antibodies JIM5 (triangle) and LM19 (circle); (b) anti-AGP antibody JIM13 (triangle), anti-arabinan antibody LM6 (circle) and anti-extensin antibody JIM20 (square); (c) anti-xyloglucan antibodies LM15 (triangle), LM24 (circle) and LM25 (square); (d) anti-callose antibody BS-400-2. Data represent the sum of CDTA and NaOH extracts. Error bars represent standard deviation (n = 9). * and ** indicate significant difference between a sample and the preceding one at p<0.05 and p<0.01, respectively.

**Figure 4:**
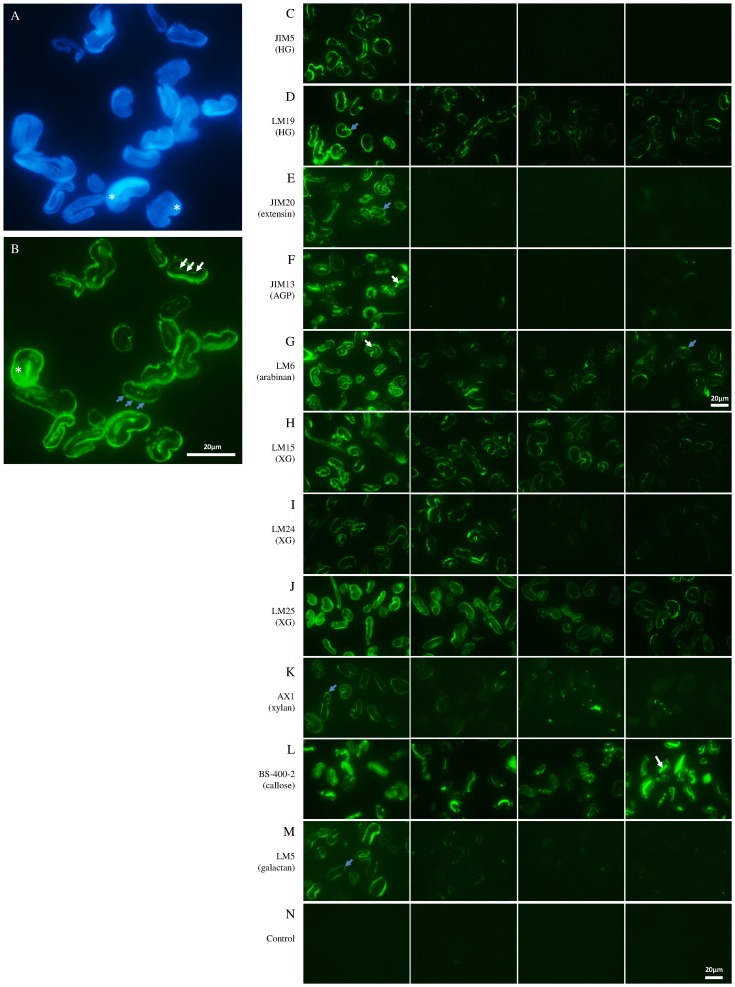
Fluorescence microscopy analysis of transverse sections through intact cotton fibres during the steps of textile processing. *In situ* immunolabeling with different antibodies (antibody used indicated on each line) on (from left to right) untreated fabric, bleached/scoured fabric, mercerized fabric and ready-to-dye fabric. A and B: respectively calcofluor staining and LM6 labeling used as fibre structure example; C to M: immunolabeling with the different probes; N: control. White and blue arrows point to examples of fluorescence detection in the fibre most inner part of the secondary cell wall and in the primary cell wall, respectively, and stars indicate the secondary cell wall. Scale bar  = 20 µm

As can be seen in table 1 and Fig. 1, bleaching/scouring is the chemical treatment that has the strongest impact on the sugar composition. During this step the monosaccharide amounts significantly decrease to approximately one third of the original amount for most of the neutral sugars (Fig. 2) and about a 3.5-fold decrease for galacturonic acid (Fig. 1). The galacturonic acid decrease is mainly associated with pectins as the main galacturonic acid containing polysaccharide is homogalacturonan (HG) [40]. This is in line with both the glycan microarray (Fig. 3A) and the immunolabeling (Fig. 4C and 4D) results, showing that HG epitopes are largely affected by the bleaching/scouring step. Depending on the HG antibody used, 80 to 90% of the signal disappears in the microarray analyses. No JIM5 (binding optimally to HG with a low level of esterification) labeling was seen in the bleached/scoured fabric while a clear staining was observed in the primary cell wall before this treatment. However, LM19 labeling (recognizing fully unesterified HG) was still observed in the samples following bleaching and scouring. This is in accordance with the microarray results that show that the signal from the JIM5 antibody is more easily removed by the treatments than the signal from the LM19 antibody. In contrast to galacturonic acid, glucuronic acid levels decrease 3-fold during the process without any of the individual steps showing a significant impact.

**Figure 5:**
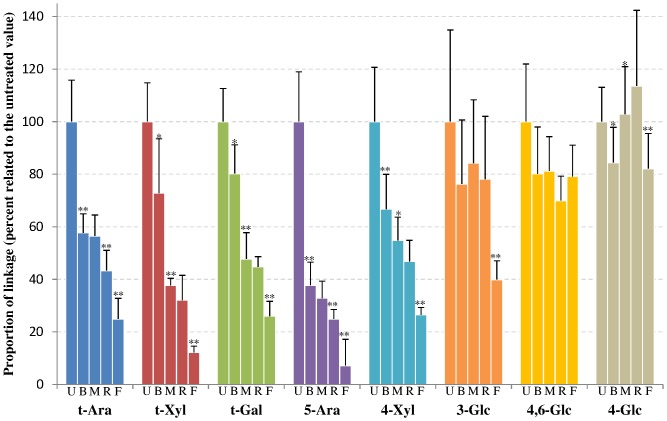
Polysaccharide linkages of powdered textile processing samples: major linkages obtained after partial methylation and hydrolysis of samples. From left to right for each linkage: U =  Untreated, B =  Bleached/scoured, M =  Mercerized, R =  Ready to dye and F =  Finished. Error bars represent standard deviation (n =  at least 3). * and ** indicate significant difference between a sample and the preceding one at p<0.05 and p<0.01, respectively.

During this bleaching/scouring step, arabinose-containing polysaccharides are also strongly affected as indicated by the levels of the individual linkages t-Ara and 5-Ara (Fig. 5) for which the decrease is 40 and 60%, respectively. Fig. 4E shows that extensin cell wall glycoproteins, that contain arabinose residues, are located both at the most inner part of the secondary cell wall and at the primary cell wall in the non-treated fabric and disappear completely in the bleached/scoured fabric. This is consistent with the results from the glycan microarray showing an 80% reduction in the extensin epitope upon bleaching/scouring (Fig. 3B). Similarly arabinogalactan-proteins (AGPs), which are located at the most inner part of the secondary cell wall, are absent from the bleached/scoured fabric (Fig. 4F). However, AGPs are less impacted in the glycan array results (Fig. 3B) with only a 40% decrease in glycan array detection compared to the complete disappearance observed by microscopy. As shown in Fig. 4B and 4G the LM6 antibody (specific for (1→5)-α-arabinan but also recognizing AGPs [41]) gives, in addition to what is likely to be the AGP signal at the most inner part of the secondary cell wall, a signal at the primary cell wall which most likely represents pectic arabinan. The bleaching/scouring step has a strong impact on the detection of the LM6 epitope, with the AGP signal from the most inner part of the secondary cell wall nearly disappearing and the arabinan signal from the primary cell wall fading as well but to a lesser extent (Fig. 4G).

Three other linkages are significantly impacted during the bleaching/scouring: t-Gal, t-Xyl, 4-Xyl (Fig. 5). The first two linkages, representative of xyloglucan, have a p-value between 0.05 and 0.01 meaning that the differences are less pronounced than for the other impacted linkages. Similarly, the binding of three different xyloglucan antibodies also show a lower impact due to the bleaching/scouring step than for most other polysaccharides (Fig. 3C). In microscopy, the LM15 signal was decreased after bleaching/scouring (Fig. 4H) whereas no obvious decrease was observed for the LM24 and LM25 antibodies (Fig. 4I and 4J). The 4-Xyl linkage is indicative of xylan and is also largely impacted during bleaching/scouring. Such a decrease in xylan is partly observed in microscopy in which AX1 labeling starts to fade during bleaching/scouring but with a more gradual fading during the subsequent processing, with almost no signal being observed in the ready-to-dye sample (Fig. 4K).

Mannose, fucose and rhamnose are also significantly impacted by bleaching/scouring as shown in Fig. 2. However, the linkages corresponding to these monosaccharides are not shown in Fig. 5 as they could not be detected. These sugars were present in lower amounts compared to galactose, xylose, and arabinose residues (as shown in Fig. 2).

### Mercerization mainly impacts xyloglucan

The second chemical processing step that has an impact on the monosaccharides is the mercerization step as shown in table 1. The decrease is less dramatic than for bleaching/scouring and is not significant for all neutral sugars except glucose. However, individual monosaccharide levels and linkage analyses show a significant decrease of fucose-, galactose- and xylose-containing polysaccharides (Figs. 2 and 5). In the linkage analysis, both t-Gal and t-Xyl have their main decrease (30 to 35%) during mercerization. Surprisingly, no significant decrease in xyloglucan was observed with the glycan profiling but a decrease does appear under the microscope for the LM24 and to a lower extent for the LM15 and LM25 xyloglucan epitopes (Fig. 4H, 4I and 4J). Indeed, xyloglucan labeling in the primary cell wall was very strong until the bleached/scoured sample and began to disappear in the mercerized sample. The LM5 signal for galactan in microscopy is weak but shows a significant decrease of intensity during both the bleaching/scouring and mercerizing steps confirming the linkage results (Fig. 4M).

### Impact of the other steps on polysaccharide composition

Treatment leading to ready-to-dye fabric led to no significant differences either for monosaccharide composition, glycan microarray or *in situ* labeling. Some differences were observed for CoMPP but, with less than 20% decrease, these differences are minor compared to the previous impacts.

A strong decrease of all linkages (except 4,6-Glc) was observed during the dyeing and finishing step (Fig. 5) and this effect was also observed for some probes in the CoMPP analysis. In contrast, this step resulted in an increased AGP signal in microarray (Fig. 3D) and an increase in arabinose and xylose monosaccharide levels.

### Callose and extracted cellulose are almost not impacted by the chemical treatments

Contrary to the polysaccharides that are impacted by bleaching/scouring or mercerization, callose is more resistant to degradation (Fig. 3D and Fig. 4L) with only 10 to 20% decrease during the whole process. This observation is also confirmed by the linkage analysis that indicates no significant difference in the level of 3-Glc between the different steps up to the ready-to-dye stage (Fig. 5). Similarly, the extracted cellulose linkage displays only minor changes during the whole process.

## Discussion

Cotton fibre is of great importance for the textile industry as it is the main material used, but it is also a good model to study cell elongation due to the ability of these single cells to expand dramatically in a highly synchronized manner [42]. Due to these particular physiological capacities, the carbohydrate composition of cotton fibre has been extensively studied during cell development [10], [43], [44], but little is known about the changes in polysaccharide composition when the mature fibre is subjected to the treatments applied during textile processing.

The mechanical treatments appeared to not induce any carbohydrate loss. This could be expected as these mechanical treatments do not affect the integrity of the fibre. Surprisingly the amount of glucose, arabinose and xylose extracted from the fibres increased after knitting. The absolute amounts of glucose detected (around 10% of the fibre weight) indicate that the glucose primarily originates from cellulose. We hypothesize that the knitting makes some domains of the cotton cellulose more easily extractable due to minor changes in the crystallinity of the fibre. These crystallinity changes could be induced by the mechanical stretching and the friction forces that occur between the yarns during knitting, similar to the impact of physical forces on cellulose crystallinity that was observed by Kumar and Kothari [45]. These changes in crystallinity might also affect the extractability of other polysaccharides leading to the observed increase of corresponding extracted monosaccharides.

The chemical treatments do result in a removal of the non-cellulosic polysaccharides from the cotton, with the impact depending on the treatment step and the specific polysaccharide being studied. Scouring is applied to make the cotton more hydrophilic for the dyeing and finishing steps through removal of pectins and waxes [16]. The drop in HG level observed after scouring/bleaching was thus expected with the hydroperoxide and alkaline treatments from this step being known to have a dramatic impact on pectins. Nevertheless, the signal that was still observed after scouring/bleaching for the LM19 HG probe indicates that some pectins might be protected by other compounds or resistant to the chemical treatments in a way that is dependent on the level of esterification. As LM19 detects fully unesterified HG, the remaining pectin is not expected to decrease the hydrophilicity, and thus not alter the suitability of the scoured cotton for further processing steps. In the same way, pectic arabinan is only partly removed during cotton processing. Glucuronic acid is most likely associated with glucuronoxylan and/or rhamnogalacturonan II (RG II). The amounts of glucuronic acid detected indicate that these polysaccharides represent only a very minor fraction of the cotton fibre that is not significantly impacted by one particular step.

Immunolabeling shows a stronger impact of bleaching/scouring on AGPs than the glycan microarray detection. Such differences between microscopy and glycan microarray profiling can be explained by an impaired accessibility of the different substrates for the *in situ* analysis and/or by the impact of the extractability of the polysaccharides for the glycan microarray.

As NaOH is commonly used to extract hemicelluloses from complex matrices [46]–[48] scouring/bleaching is expected to extract most of the hemicellulose. Unexpectedly, xyloglucan appeared to resist the bleaching/scouring step more than the other hemicelluloses. This was evidenced by the three xyloglucan probes used for the CoMPP and microscopy experiments. These three probes showed different patterns of detection that are explained by the different recognition of xyloglucan epitopes by the antibodies: XXXG for LM15 [38], XXLG and XLLG for LM24 and LM25 [33]. This means that depending on the side chains present on the xyloglucan backbone, the polysaccharide will either be more or less sensitive to the chemicals or more strongly associated with other sodium hydroxide-resistant polysaccharides [46], [47] such as cellulose or callose. It should be noted as well that the 4,6-Glc linkage was largely unaffected by the chemical treatments but this result should be taken with caution as it can be contaminated by partial derivatization of cellulose, as this latter compound may overwhelm the derivatizing agents [26]. It was shown in previous studies that alkali treatments remove hemicelluloses, convert cellulose I to cellulose II and decrease cellulose crystallinity [49]. It was also shown more specifically that mercerization, that consists of a strong sodium hydroxide treatment of the fabric, affects the crystallinity of cotton cellulose [39], [50]. As the bleaching/scouring step also includes a (weaker) sodium hydroxide treatment, most of the hemicelluloses are already removed to a large extent by that step and are not strongly affected any further by mercerization. As most of the xyloglucan is not removed during the scouring/bleaching step, it is likely to be closely associated with the fibre cellulose. As a consequence, the change in cellulose structure induced by the mercerization would affect the association of xyloglucan with the cellulose and make this compound more vulnerable to the sodium hydroxide.

Amongst all the polysaccharides (excluding cellulose) observed during this study, callose appeared to be the most resistant to the chemical treatments but this is not surprising as it was already shown by Kohler *et al.* [51] that callose resists alkali extractions.

For the last step of the textile processing, leading to the finished fabric, all results apparently point towards a strong impact on the carbohydrate composition. However, these results should be interpreted with care as the dyes and the finishing agents are expected to react with the hydroxyl functions of the polysaccharides. Indeed, the dyes used in this study are reactive dyes, which react with the free hydroxyl groups of osidic units [52]. If an osidic unit reacts with a dye molecule, the hydroxyl group that was free before will be occupied and cannot be methylated nor acetylated anymore. This osidic unit is then no longer identified as the original linkage because it is “masked” by the dye. The dramatic decrease in linkages observed at this step is thus probably not linked to the disappearance of the corresponding polysaccharides. The implication for nearly all linkages would mean that most polysaccharides are targeted by the dye and/or the finishing products. The reaction with the dye or finishing products may also result in a masking of specific epitopes and might therefore also explain the decrease observed in glycan microarray signals for HG (LM19), and xyloglucan (LM15 and LM24). However, dyeing and finishing also result in an increased microarray signal for callose and AGPs and an increased arabinose and xylose content. A possible explanation for this observation might be that the reaction with the dye or finishing molecule separates the concerned polysaccharides more from each other within the fibre and makes them more accessible for detection and extraction.

## Conclusions

Based on these observations, the different components of the cotton fibre can be classified according to their responses to the chemical treatments used during fibre/textile processing. Interestingly, non-cellulosic polysaccharides are not completely eliminated during textile processing. HG, AGP, extensin and arabinan are strongly affected by the bleaching/scouring step with little or none of these glycans detectable at the end of the process. Xylan and xyloglucan are more resistant but are still largely impacted by both bleaching/scouring and mercerization. Amongst the xyloglucan some forms seem to be more resistant than others. Callose is resistant to the different chemical treatments with most still being present at the end of processing. These experiments shed some light on the fate of non-cellulosic polysaccharides during textile processing. This brings new possibilities for future functionalization of cotton by specifically targeting these non-cellulosic polysaccharides and for optimization of cotton treatments. It also shows that hemicelluloses can be resistant to harsh treatments possibly depending on their molecular environment as some of these hemicelluloses within the fibre structure survived the textile processing treatments.

## Supporting Information

S1 Table **Comprehensive microarray polymer profiling of powdered textile processing samples.** The analysis was conducted as described by Singh et al., 2009 [5] and in the CoMPP description paragraph in the material and methods. Values have been individually rescaled for each antibody to a maximum of 100 and colored from grey to green accordingly to the values.
(TIF)
