# Supplementary figures and images for: Non-Cellulosic Polysaccharides from Cotton Fibre Are Differently Impacted by Textile Processing
Source: PLoS One. 2014 Dec 17;9(12):e115150. doi: 10.1371/journal.pone.0115150 (PMC4269390; doi:10.1371/journal.pone.0115150)

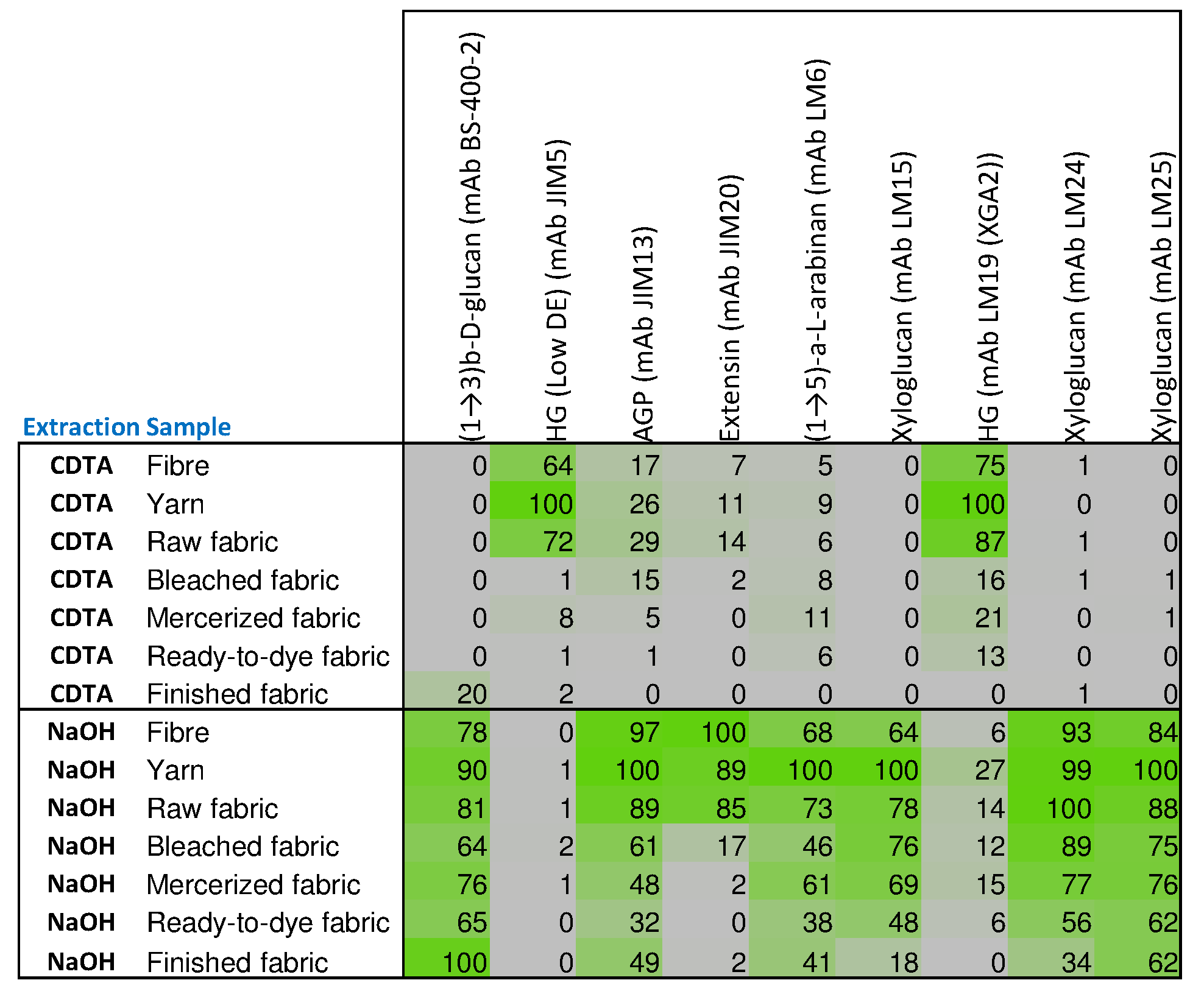

Supplement: S1 Table — Comprehensive microarray polymer profiling of powdered textile processing samples. The analysis was conducted as described by Singh et al., 2009 [5] and in the CoMPP description paragraph in the material and methods. Values have been individually rescaled for each antibody to a maximum of 100 and colored from grey to green accordingly to the values. (TIF) [file pone.0115150.s001.tif]
